# Evaluation of different time domain peak models using extreme learning machine-based peak detection for EEG signal

**DOI:** 10.1186/s40064-016-2697-0

**Published:** 2016-07-11

**Authors:** Asrul Adam, Zuwairie Ibrahim, Norrima Mokhtar, Mohd Ibrahim Shapiai, Paul Cumming, Marizan Mubin

**Affiliations:** Applied Control and Robotics (ACR) Laboratory, Department of Electrical Engineering, Faculty of Engineering, University of Malaya, 50603 Kuala Lumpur, Malaysia; Faculty of Electrical and Electronic Engineering, Universiti Malaysia Pahang, 26600 Pekan, Pahang Malaysia; Malaysia-Japan International Institute of Technology, Universiti Teknologi Malaysia Kuala Lumpur, Jalan Semarak, 54100 Kuala Lumpur, Malaysia; School of Psychology and Counseling, Queensland University of Technology, and QIMR Berghofer, Brisbane, Australia

**Keywords:** Extreme learning machines (ELM), Electroencephalogram (EEG), Peak detection algorithm, Peak model, Pattern recognition

## Abstract

Various peak models have been introduced to detect and analyze peaks in the time domain analysis of electroencephalogram (EEG) signals. In general, peak model in the time domain analysis consists of a set of signal parameters, such as amplitude, width, and slope. Models including those proposed by Dumpala, Acir, Liu, and Dingle are routinely used to detect peaks in EEG signals acquired in clinical studies of epilepsy or eye blink. The optimal peak model is the most reliable peak detection performance in a particular application. A fair measure of performance of different models requires a common and unbiased platform. In this study, we evaluate the performance of the four different peak models using the extreme learning machine (ELM)-based peak detection algorithm. We found that the Dingle model gave the best performance, with 72 % accuracy in the analysis of real EEG data. Statistical analysis conferred that the Dingle model afforded significantly better mean testing accuracy than did the Acir and Liu models, which were in the range 37–52 %. Meanwhile, the Dingle model has no significant difference compared to Dumpala model.

## Background

Peak detection algorithms are prominently used for event classification in various physiological signals such as in electroencephalograms (EEG) for diagnosing epilepsy (Acir 2005), photoplethysmograms (PPG) for monitoring heart rate (Elgendi et al. 2013), and in EEG (Adam et al. 2014b) or electrooculograms (EOG) in the particular application of tracking eye gaze events (Barea et al. 2012). In all of these common applications, peak detection is commonly the first step in signal processing. For example, semi-automatic diagnosis of epilepsy can be based on the frequency of peaks detected in the EEG recording during a given time interval. A similar approach is used for identifying eye blink events, a frequent source of interference in EEG recordings.

Detecting a peak indicative of a particular event in the EEG signal is challenging due to the non-stationary nature of the signal relative to the baseline amplitude, time, and different user. A signal peak identified as a point of highest signal amplitude lying between two associated valley points, which hold a local minimum value. Any single peak is described by a number of signal parameters, including amplitude, width, and slope. Based on those parameters, a number of peak features can be calculated in the temporal domain, such as peak-to-peak amplitudes at the first half wave, peak width, ascending peak slopes at the first half wave, and descending peak slope at the second half wave. The ensemble of these peak features serves to detect the peak in various applications. However, the high variation of calculated peak features in real EEG data, which typically contain several types of noise, can interfere with correct peak detection and degrade performance.

Typically, a peak detection algorithm consists of combination of selected features from their peak model and subsequent computational processes, such as classification. Based on the literature, Dumpala et al. (1982) used a defined peak model, and then introduced a classification process to detect a peak signal in the analysis of gastric electrical activity. Dingle et al. (1993); Liu et al. (2002); Acir et al. (2005); Acir 2005); Liu et al. (2013) also used the defined peak models and different classification processes to detect peaks in EEG signal with epileptiform activity. The classifiers that have hitherto been used for signal peak detection include rule-based (Dumpala et al. 1982; Adam et al. 2015; Dingle et al. 1993), AdaBoost (Liu et al. 2002), radial basis function network (Acir et al. 2005), support vector machine (SVM) (Liu et al. 2013), radial basis support vector machine (Acir et al. 2005), artificial neural network (ANN) (Liu et al. 2002), and expert system (Liu et al. 2002; Dingle et al. 1993). In general, utilization of a peak detection algorithm provides the best performance in various applications. However, the various algorithms have used different peak models and different classification approaches combined in a particular peak detection algorithm. Moreover, to the best of our knowledge, there are very reports comparing the performance of different peak models using the same classification platform, such as a rule-based classifier (Adam et al. 2014a, 2014b, 2015). The existing methods tend to have poor performance with peak models defining many peak features, for example, the detection performance declined to nil when the classifier employed all 11 features from Liu model (Adam et al. 2014b).

For fair evaluation of the detection performance of different EEG signal peak models, they must be assessed using a common classification method. Therefore, in this study we used the extreme learning machine (ELM) method as a common classifier for the peak detection algorithm to evaluate the performance in association with four different peak models in time domain analysis, namely the models of Dumpala, Acir, Liu, and Dingle. The four representative peak models were on the basis of their proven utility in various physiological signal applications. We used an ELM since it provides very fast learning speed, generalized performance, learning without iterative tuning, and minimal requirement for user intervention. ELM also employs as alternative method to resolve the shortcoming of existing studies which have poor performance to peak model with many peak features. Hence, the present study aims to determine the best peak model in time domain analysis for EEG-based horizontal eye movement signal application using an advantageous common classification platform.

## Methods

### EEG signal peak detection algorithm

**Fig. 1:**
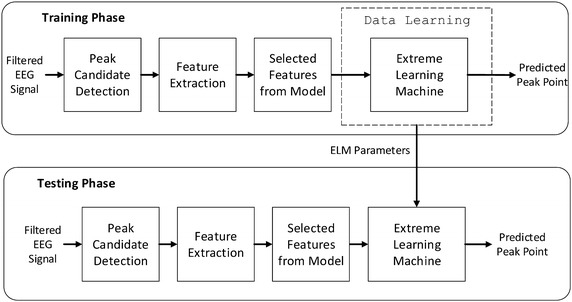
Training and testing phases of EEG signal peak detection

The training and testing phases of the EEG signal peak detection algorithm are shown in Fig. 1. The training and testing data that used in this study were collected using two channel EEG recordings from 20 voluntary subjects. In the first stage of peak detection, the training and testing EEG signals must be filtered as input to the algorithm, upon selection of the desired peak model. The training phase of the algorithm involves several processes, namely including peak candidate detection, feature extraction, with definition of model-specific features, and then classification process. The estimation process is performed during this phase to train the network for adjusting the ELM parameters using the learning algorithm of the ELM classifier. In the testing phase, the algorithm follows the same series of processes, and the ELM parameters first determined in the training phase are used in the classification process of the testing phase. The final output of the training and testing phase are the predicted peak points and non-peak points from the identified peak candidates.

### Peak candidate detection

The first process of the detection algorithm is to determine candidate peaks. This process is used to assign the peaks into two groups, true non-peaks and true peaks. The group of true peaks is reconsidered to consist of candidate peaks, which can further classified into true non-peaks and true peaks using the ELM classifier. One advantage of determining candidate peaks process is that it reduces the number of input samples required for the ELM classifier, such that the computational time in the training and testing phases is minimized.

**Fig. 2:**
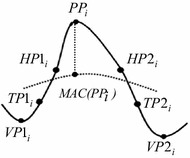
The eight points of peak model

Determining the local maxima (peak points) and minima (valley points), the first process in determining a candidate peak, can be performed using an algorithm developed by Billauer (2012). The subsequent process of detecting a peak candidate is as follows: By considering a discrete-time signal, *x*(*I*) of *L* points, the *i*th candidate peak point, *PP*_*i*_, is identified using the three-point sliding window method (Dumpala et al. 1982). The three selected points are denoted as *x*(*I*−1), *x*(*I*) and *x*(*I* + 1) for *I* = 1, 2, 3, …,*L*. A candidate peak point is identified when *x*(*PP*_*i*_−1) < *x*(*PP*_*i*_) > *x*(*PP*_*i*_ + 1) and two associated valley points, *VP*1_*i*_ and *VP*2_*i*_ lie on either side of the peak, as shown in Fig. 2. The valley points are defined when *x*(*VP*1_*i*_−1) > *x*(*VP*1_*i*_) < *x*(*VP*1_*i*_ + 1) and *x*(*VP*2_*i*_−1) > *x*(*VP*2_*i*_) < *x*(*VP*2_*i*_ + 1).

### Feature extraction

The features of a peak candidate are calculated based on the eight points shown in Fig. 2. The set of points consists of the *i*th candidate peak point, *PP*_*i*_, the two associated valley points, *VP*1_*i*_ and *VP*2_*i*_, the half point at first half wave (*HP*1_*i*_), the half point at second half wave (*HP*2_*i*_), the turning point at first half wave (*TP*1_*i*_), the turning point at second half wave *(TP*2_*i*_), and the moving average curve point (*MAC(PP*_*i*_)). The half point at first half wave can be defined as the point in slope located in the middle between the*PP*_*i*_ and *VP*1_*i*_ points while the half point at the second half wave as the point in slope based in the midst between the *PP*_*i*_ and *VP*2_*i*_ points. The *MAC(PPi*) point is located at the intersection between the *PP*_*i*_ and *MAC(PPi*) points. The window length of the moving averaging is 100 sampling points.

Based on signal parameters, the features of a peak candidate can be categorized into three groups, namely amplitude, width, and slope. There are five different amplitudes, seven different widths, and four different slopes that can be calculated based on the eight defined points, resulting in a total of 16 features, which can be defined as follows:

1. The peak-to-peak amplitude at the first half wave, *f*_1_, is the peak amplitude between the magnitude of the peak and the magnitude of the valley of the first half wave, as denoted by.
  1 $$ f_{1} = \left| {y\left( {PP_{i} } \right) - y\left( {VP1_{i} } \right)} \right| $$
2. The peak-to-peak amplitude at the second half wave, *f*_2_, is the peak amplitude between the magnitude of the peak and the magnitude of the valley of the second half wave, and is defined as
  2 $$ f_{2} = \left| {y\left( {PP_{i} } \right) - y\left( {VP2_{i} } \right)} \right| $$
3. The turning point amplitude at the first half wave, *f*_3_, is the peak amplitude between the magnitude of the peak and the magnitude of the turning point at the first half-wave. The turning point is defined as the point where the slope decreases more than 50 % compared to the slope of the preceding point. The equation for *f*_3_ is as follows:
  3 $$ f_{3} = \left| {y\left( {PP_{i} } \right) - y\left( {TP1_{i} } \right)} \right| $$
4. The turning point amplitude at the second half wave, *f*_4_, is the peak amplitude between the magnitude of the peak and the magnitude of the turning point at the second half wave, and is defined as
  4 $$ f_{4} = \left| {y\left( {PP_{i} } \right) - y\left( {TP2_{i} } \right)} \right| $$
5. The moving average amplitude, *f*_5_, is the peak amplitude between the magnitude of the peak and the magnitude of the moving average, and is defined as
  5 $$ f_{5} = \left| {y\left( {PP_{i} } \right) - y(MAC\left( {PP_{i} } \right))} \right| $$
6. The peak width, *f*_6_, is the peak width between the valley point of the first half point and the valley point of the second half wave, and is defined as
  6 $$ f_{6} = \left| {x(VP1_{i} ) - x(VP2_{i} )} \right| $$
7. The first half wave width, *f*_7_, is the peak width between the peak point and the valley point of the first half wave, and is defined as
  7 $$ f_{7} = \left| {x(PP_{i} ) - x(VP1_{i} )} \right| $$
8. The second half wave width, *f*_8_, is the peak width between the peak point and the valley point of the second half wave, and is defined as
  8 $$ f_{8} = \left| {x(PP_{i} ) - x(VP2_{i} )} \right| $$
9. The turning point width, *f*_9_, is the peak width between the turning point at the first half wave and the turning point at the second half wave, and is defined as
  9 $$ f_{9} = \left| {x(TP1_{i} ) - x(TP2_{i} )} \right| $$
10. The first half-wave turning point width, *f*_10_, is the peak width between the turning point at the first half-wave and the peak point, and is defined as
  10 $$ f_{10} = \left| {x(PP_{i} ) - x(TP1_{i} )} \right| $$
11. The second half wave turning point width, *f*_11_, is the peak width between the turning point at the second half-wave and the peak point, and is defined as
  11 $$ f_{11} = \left| {x(PP_{i} ) - x(TP2_{i} )} \right| $$
12. The half point width, *f*_12_, is the peak width between the half point of the first half-wave and the half point of the second half-wave, and is defined as
  12 $$ f_{12} = \left| {x(HP1_{i} ) - x(HP2_{i} )} \right| $$
13. The peak slope at the first half wave, *f*_13_, is the maximal slope between the peak point and the valley point of the first half wave, and is defined as
  13 $$ f_{13} = \left| {\frac{{y\left( {PP_{i} } \right) - y\left( {VP1_{i} } \right)}}{{x(PP)_{i} - x(VP1_{i} )}}} \right| $$
14. The peak slope at the second half-wave, *f*_14_, is the peak slope between the peak point and the valley point of the second half wave, and is defined as
  14 $$ f_{14} = \left| {\frac{{y\left( {PP_{i} } \right) - y\left( {VP2_{i} } \right)}}{{x(PP_{i} ) - x(VP2_{i} )}}} \right| $$
15. The turning point slope at the first half-wave, *f*_15_, is the peak slope between the peak point and the turning point of the first half-wave, and is defined as
  15 $$ f_{15} = \left| {\frac{{y\left( {PP_{i} } \right) - y\left( {TP1_{i} } \right)}}{{x(PP_{i} ) - x(TP1_{i} )}}} \right| $$
16. The turning point slope at the second half wave, *f*_16_, is the peak slope between the peak point and the turning point of the second half-wave, and is defined as
  16 $$ f_{16} = \left| {\frac{{y\left( {PP_{i} } \right) - y\left( {TP2_{i} } \right)}}{{x(PP_{i} ) - x(TP2_{i} )}}} \right| $$

From these 16 features, Dumpala et al. (1982) introduced a peak model that uses the four most salient features, *f*_1_, *f*_6_, *f*_13_, and *f*_14_. Additional features defining peak amplitude, *f*_2,_ and two features of peak width, *f*_7_ and *f*_8,_ were introduced by Acir et al. (2005); Acir and Guzeli (2004). As pointed out in Acir et al. (2005), the defined features are interrelated to the characteristic of peak in epilepsy events. There are highlighted three characteristics as follows; (1) the ascending peak slopes at the first half wave, and descending peak slope at the second half wave are relatively large and smooth (2) the top of the peak is sharp, and (3) the peak width is always between 20 and 70 ms. The peak width can be calculated by the sum of the first half wave and second half wave peak width. Dumpala et al. (1982) and Acir et al. (2005) used the same similar definition of the peak slopes, i.e. *f*_13_ and *f*_14_. The peak model of Liu et al. (2002) entailed a total of 11 features, consisting of four amplitudes (*f*_1_, *f*_2_, *f*_3_, and *f*_4_), three widths (*f*_6_, *f*_9_, *f*_12_), and four slopes (*f*_13_, *f*_14_, *f*_15_, *f*_16_). Finally, the peak model introduced by Dingle et al. (1993) consists of four features (*f*_5_, *f*_6_, *f*_13_, *f*_14_). The different peak models and their sets of features are listed in Table 1.

**Table 1:** List of different peak models and sets of features

Peak model	Set of features	Number of features
Dumpala et al. (1982)	*f*_1_, *f*_6_, *f*_13_, *f*_14_	4
Dingle et al. (1993)	*f*_5_, *f*_6_, *f*_13_, *f*_14_	4
Acir et al. (2005); Acir (2005); Acir and Guzeli (2004)	*f*_1_, *f*_2_, *f*_7_, *f*_8_, *f*_13_, *f*_14_	6
Liu et al. (2002)	*f*_1_, *f*_2_, *f*_3_, *f*_4_, *f*_6_, *f*_9_, *f*_12_, *f*_13_, *f*_14_, *f*_15_, *f*_16_	11

### Extreme learning machine classifier

Extreme learning machine is a new approach to machine learning involving a single layer feedforward neural network (SLFN). The introduction of ELM by Huang et al. (2004) has been followed by several variants (Balasundaram et al. 2014; Huang et al. 2012). ELM has already been used with success for various event classifications of EEG signals (Song and Zhang 2013; Shi and Lu 2013; Yuan et al. 2012; Song et al. 2012; Yuan et al. 2011; Liang et al. 2006). The main advantage present by ELM is the fast computation of the learning method compared to conventional ANN learning, since ELM training dispenses with time-consuming iterative tuning.

**Fig. 3:**
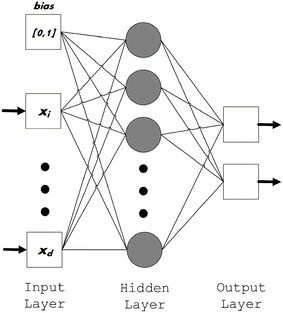
ELM architecture

The architecture of an ELM is shown in Fig. 3. The network consists of three layers, i.e. the input, hidden, and output layers. Between the input and hidden layers are the input weights, and between the hidden and output layers are the output weights. The training process of an ELM proceeds in three stages. In the first stage, the input weights are assigned randomly between −1 and 1, and the biases in the hidden layer are assigned randomly between 0 and 1. Both of these parameters remain fixed during the training process. Afterward, the output matrix of the hidden layer, *H*, is calculated as follows:

17 $$ H = \left[ {\begin{array}{*{20}c} {h(x_{1} )} \\ \vdots \\ {h(x_{N} )} \\ \end{array} } \right] = \left[ {\begin{array}{*{20}c} {g\left( {\sum\nolimits_{i = 1}^{d} {a_{i1} x_{1i} + b_{1} } } \right)} & \cdots & {g\left( {\sum\nolimits_{i = 1}^{d} {a_{iL} x_{1i} + b_{L} } } \right)} \\ \vdots & \ddots & \vdots \\ {g\left( {\sum\nolimits_{i = 1}^{d} {a_{i1} x_{Ni} + b_{1} } } \right)} & \cdots & {g\left( {\sum\nolimits_{i = 1}^{d} {a_{iL} x_{Ni} + b_{L} } } \right)} \\ \end{array} } \right]_{N \times L} $$

where *g* is an activation function of the hidden neuron, *x* is the *N* × *L* matrix of inputs, *a* is the *d* × *L* matrix of random input weights, *b* is the 1 × *L* matrix of random biases in the hidden layer, *N* is an arbitrary number of distinct samples, *L* is the number of hidden neurons, and *d* is the number of inputs or features The *i*th column of *H* is the output of the *i*th hidden neuron with respect to inputs *x*_1_, *x*_2_, until *x*_*d*_.

The ELM can be represented as a linear system, which is mathematically modeled as

18 $$ H\beta = T $$

where *β* is the *L* × *m* matrix of output weights, *T* is the *N* × *m* matrix of target outputs, and *m* is the number of output neurons. The *β* and *T* matrixes are denoted as

19 $$ \beta = \left[ {\begin{array}{*{20}c} {\beta_{1}^{\rm T} } \\ \vdots \\ {\beta_{L}^{\rm T} } \\ \end{array} } \right]_{L \times m} $$

and

20 $$ T = \left[ {\begin{array}{*{20}c} {t_{1}^{\rm T} } \\ \vdots \\ {t_{N}^{\rm T} } \\ \end{array} } \right]_{N \times m} $$

respectively. To determine the least square solution, *β,* of the linear system *Hβ* = *T*, the minimum norm least-squares solution is computed as follows:

21 $$ \left\| {H\left( {a_{1} , \cdots ,a_{L} ,b_{1} , \cdots ,b_{L} } \right)\beta - T} \right\| = \mathop {\hbox{min} }\limits_{\beta } \left\| {H\left( {a_{1} , \cdots ,a_{L} ,b_{1} , \cdots ,b_{L} } \right)\beta - T} \right\| $$

It is well known that the smallest norm least-squares solution of Eq. 21 is

22 $$ \beta = (H^{\rm T} H)^{ - 1} H^{\rm T} T = H^{ + } T $$

where *H*^+^ is the Moore–Penrose pseudo-inverse of *H*. The three training stages of the ELM classifier are summarized as follows:

Stage 1: Randomly assign the input weights, *a*_*i*_ and biases in the hidden neurons, *b*_*i*_
Stage 2: Calculate the output matrix of the hidden layer, *H*
Stage 3: Calculate the output weights, *β* = *H*^+^*T*.

The output function of the ELM classifier of a given unknown sample *x* is

23 $$ f(x) = h(x)\beta $$

In the output layer, two neurons are used in the network to classify the output into two classes (output): class 1 and class 0. For the two classes (*m* > 1), the predicted class label is the *i*th number of the output neurons, which is the maximum value of the output neuron (Huang et al. 2012). The predicted class label of a given unknown sample *x* is defined as follows.

24 $$ label(x) = \mathop {\arg \hbox{max} f_{i} (x)}\limits_{{i \in \left\{ {1, \ldots ,m} \right\}}} $$

We evaluate the performance of our ELM classifier based on the *G*_*mean*_ (Guo et al. 2008), which is calculated as follows:

25 $$ TPR = \frac{TP}{TP + FN} $$

26 $$ TNR = \frac{TN}{TN + FP} $$

27 $$ G_{mean} = \sqrt {TPR \times TNR} $$

where a true peak (*TP*) is the correctly-detected peak point of the peak candidate, a true non-peak (*TN*) is the correctly-detected non-peak point of the peak candidate, a false peak (*FP*) is the wrongly-detected non-peak point of a peak candidate, a false non-peak (*FN*) is a wrongly-detected peak point of a peak candidate, *TPR* is the true peak rate, and *TNR* is the true non-peak rate.

### Experimental setup and protocols

Each experiment is conducted in 30 independent runs. The first 50 % of the filtered EEG signal was used as training data, and the remaining 50 % as testing data.

The parameter settings of the ELM classifier are shown in Table 2. The number of hidden neuron was selected using a trial and error method, which was set to 500. The sigmoid [−1, 1] was used as an activation function in the hidden layer for the purpose of normalization, whereas a linear function was located on each neuron in the output layer. Other settings for the ELM classifier, such as the number of neurons in the input layer are dependent on the number of selected features of a particular peak model. The number of output neurons was set to 2 which it used maximum argument as indicated in Eq. 24 for choosing the ELM output. We note that the input weights and the biases remained fixed during the training, but the values of these two ELM parameters are randomly assigned for each of 30 runs.

**Table 2:** Parameter settings of ELM

Parameters	Value
Number of neurons in the hidden layer	500
Biases in the hidden layer	Random [0, 1]
Activation function in the hidden layer	Sigmoid [−1, 1]
Activation function in the output layer	Linear function
Number of neurons in the input layer	Depends on a number of features
Number of neurons in the output layer	2

This experimental protocol was approved by the medical ethics committee of the University of Malaya Medical Centre. All subjects signed informed consent forms in advance.

The filtered EEG signals in this study were obtained in the Applied Control and Robotic (ACR) Laboratory, Department of Electrical Engineering, Faculty of Engineering, University of Malaya, Malaysia. Twenty healthy subjects (10 males and 10 females, aged 20–40 years), who are undergraduate and postgraduate students in the Faculty of Engineering, volunteered to participate in these data collection sessions. Filtered EEG signal recordings were obtained using the g.MOBIlab portable biological signal acquisition system. The scalp electrodes were arranged using the 10–20 international electrode placement system. The EEG signal was recorded from the C3 and C4 channels, with the signal of channel CZ used as a reference. The ground electrode was located on the FPz channel, such that a total of only four electrodes were used. The sampling frequency was set to 256 Hz. The electrodes from the C3 and C4 channels are positioned for detecting EEG peaks associated with the brain response of commanded horizontal eye gaze direction. We used the C3, C4, and CZ channels are used because of they have relatively little less contamination from EEG artifacts due to eye blinking (Klados et al. 2011).

All subjects had been instructed to get a good rest the night before the data collection session, so as to ensure full focus during the EEG recordings. The subjects were told to prepare for the external voice cue within up to 4 s. Appearance of the cue is voice command or verbal reminder for the subject to move his eyes initially forward fixation to the left or to the right. At exactly 5 s from the beginning session, the external voice cue appears randomly instructing the subject to shift gaze to the left or right direction, and hold the new eye position from 5 until 10 s, which is the end of the EEG recording. The eye gaze directions that produce a number of peaks in the signal on channels C3 and C4 are archived as raw data for analysis.

**Fig. 4:**
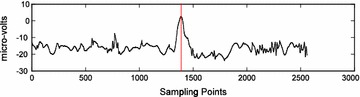
Filtered EEG-based eye movement signal (one peak point per signal)

Figure 4 shows a representative case of filtered EEG signals that are labeled as eye movement signals. The dotted red vertical lines show the actual peak point locations, as assigned by a researcher. The eye movement signal consists of 20 signals for channel C3, 20 signals for channel C4, for duration of 10-s per signal, recorded at 256 Hz for a total of 2560 sampling points per signal. Furthermore, each signal contains one known peak point location, where the known peak pattern represents the eye gaze direction, either to the left or to the right.

## Experimental results and discussion

Four different outcome measures were used in the experiments: the average *G*_*mean*_, the maximum *G*_*mean*_, the minimum *G*_*mean*_, and the standard deviation (STDEV). Additionally, the statistical comparisons of the average test accuracy among the four models are evaluated using Friedman’s test.

The results of training and testing performance based on the four different measurements (end-points) for the peak model with all 16 features are shown in Table 3. For training performance, the average, maximum, minimum, and STDEV values were 88.3, 94.9, 80.6, and 3.6 %, respectively, whereas for testing performance, the average, maximum, minimum, and STDEV values were 36.9, 58.1, 0, and 11.8 %, respectively. The minimum testing performance of 0 % indicates that the classifier was unable to correctly detect even one peak.

**Table 3:** Peak detection training and testing performance using all features

Measurement	Training (%)	Testing (%)
Average	88.3	36.9
Maximum	94.9	58.1
Minimum	80.6	0
STDEV	3.6	11.8

Next, the training and testing performance based on the four different measurements for each peak model are shown in Table 4, the training performance average, maximum, minimum, and STDEV values are 84.7, 86.6, 83.7 and 1.4 % for Dumpala peak model, whereas the corresponding results were 88.3, 89.4, 86.6 and 1.4 % for Acir peak model, 78.9, 83.7, 74.1 and 2.6 % for Liu peak model, and 99.5, 100, 97.4 and 0.9 %, for Dingle peak model. The performance of Dingle peak model was clearly superior to that of the other peak models.

**Table 4:** Peak detection training and testing performance for each peak model

Peak model	Training (%)				Testing (%)			
	Average	Max	Min	SD	Average	Max	Min	SD
Dumpala	84.7	86.6	83.7	1.4	70.1	82.6	51.6	6.7
Acir	88.3	89.4	86.6	1.4	36.9	62.6	0	11.9
Liu	78.9	83.7	74.1	2.6	52.1	71.8	37.2	7.9
Dingle	99.5	100	97.4	0.9	71.7	89.2	57.1	6.9

The testing performance on average, maximum, minimum, and STDEV values are 70.1, 82.6, 51.6 and 6.7 %, respectively, for Dumpala peak model; 36.9, 62.6, 0 and 11.9 %, respectively, for Acir peak model; 51.1, 71.8, 37.2 and 7.9 %, respectively, for Liu peak model and 71.7, 89.2, 57.1 and 6.9 %, respectively, for Dingle peak model. As with the training data, performance of Dingle peak model with the test data was superior to that of the other peak models. As with the training set, results with Acir method for the test data showed that the classifier is unable to correctly predict all the true peaks occurring at the particular time when TP is equal to zero. Thus, *G*_*mean*_ becomes zero even though the TN value is non-zero.

Sensitivity is the percentage of true peak rate recovered while specificity is the percentage of true non-peak rate. The overall sensitivity and specificity values for the testing performance are shown in Table 5. The results in Table 5 show that sensitivity is significantly lower than 30 % for the Acir and Liu models. Dumpala and Dingle peak models performed best, with sensitivity of 55 % and specificity exceeding 99 %. Overall, the sensitivity of the four peak models is lower than their specificity, thus resulting in a large amount of false non-peak. The four peak models return many false non-peaks due to several contributing factors such the collected data is affected by various noises and the peak features have a large different value from one subject to another subject. These factors are the cause to the high variation of peak features of the four peak models. In this case, the NNRW classifier has performed best for classifying the non-peak features than peak features.

**Table 5:** Sensitivity and specificity testing performance for each peak model

Peak model	Sensitivity (%) = $\frac{TP}{TP + FN} \times 100$	Specificity (%) = $\frac{TN}{TN + FP} \times 100$
Dumpala	57.2	99.6
Acir	18	99.9
Liu	28.3	99.7
Dingle	55	99.7

The comparison of the average test accuracy between the four peak models is extended using Friedman’s test for statistical analysis. The analysis searches for a significant difference in the average testing accuracies between the peak models with *p* value lower than threshold of 0.01. The average rankings of Friedman’s test (Table 6) show best results for Dingle peak model, followed by the Dumpala, Liu, and Acir peak models. Post-hoc analysis of Friedman’s test results are based on the Holm-Bonferroni method using two difference confidence intervals, α = 0.05 and α = 0.10 as shown in Table 7. Both Friedman’s test and the Holm-Bonferroni post hoc analysis are conducted using the KEEL software tool (Alcala-Fdez et al. 2009). The post hoc results in Table 7 show similar rank orders for α = 0.05 and α = 0.10, where Dingle peak model offers test accuracies significantly better than do Acir and Liu peak models, but was not superior to without any significant compared to Dumpala peak model.

**Table 6:** Average ranking of Friedman’s test with *p* < 0.01

Peak model	Average ranking	Rank
Dumpala	1.5667	2
Acir	3.7	4
Liu	3.2333	3
Dingle	1.5	1

**Table 7:** Post-hoc analysis for Friedman’s test

*i*	Condition	α = 0.05		α = 0.10	
		*p*	Holm	*p*	Holm
6	Acir vs. Dingle	0.000001	0.00833	0.000001	0.01667
5	Dumpala vs. Acir	0.000001	0.01	0.000001	0.02
4	Liu vs. Dingle	0.000001	0.0125	0.000001	0.025
3	Dumpala vs. Liu	0.000001	0.01667	0.000001	0.03333
2	Acir vs. Liu	0.161513	0.025	0.161513	0.05
1	Dumpala vs. Dingle	0.841481	0.05	0.841481	0.1

## Conclusions and future work

In this study, we applied ELM-based peak detection to two-lead EEG signals recorded from 20 healthy subjects instructed to direct their horizontal gaze in response to a voice cue. The data was used to evaluate the performance of four different peak detection models. The various event-related EEG peaks were analyzed through a series of processes, i.e. peak candidate detection, feature extraction, and classification. The four peak models considered in this study are representative of typical EEG studies in the literature (Dumpala et al. 1982; Acir et al. 2005; Acir 2005; Dingle et al. 1993; Liu et al. 2002), all of which entail initial extraction of 16 peak features. Each of the four peak models was selected in turn before the execution of experiments using the ELM as a common classification algorithm. ELM has been tested on more than forty benchmark data sets. Also, ELM has been proven by experimental results that achieved similar or better generalized performance compared to SVM and least square support vector machine (LS-SVM) for two-class classification (Huang et al. 2012). We find Dingle peak model to be the best for reliably detecting voluntary horizontal eye movement signal peaks, delivering a mean performance of 99.5 % for the training set and 71.7 % for the testing set. The testing performance needs to be improved by reconsidering the selection of peak features among 16 features and exploring other variant of ELM classifiers. Furthermore, the results in Table 4 also indicate that Dingle peak model to be a good generalized model, as revealed by the highest classification rate of the minimum testing result at 57.1 %. Additionally, Friedman’s test confirms that Dingle peak model offers significant better average test accuracy than those of Acir and Liu models.

This study also observes that defining more peak features on model is not guarantee in producing better accuracy on EEG-based horizontal eye movement signal application. As shown in the results in Table 3, the mean of testing accuracy only can achieve at 36.9 %. However, determining the optimal model from the selected features associated with the advantageous of common classification platform is the best approach to gain the accuracy of detection performance.

Results of this study may be applicable in many contexts characterized by the general problem of signal detection, including, such as medical diagnostics, human–machine interface (HMI), brain-computer interface (BCI), and harmonic detection in digital and audio signal processing. For example, an EEG peak in the frontal eye field associated with a change of horizontal eye gaze direction could be translated to the direction of cursor movement in BCI applications, which might be useful for patients with locked-in syndrome or other disabilities (Belkacem et al. 2014). This approach might also be translatable for EEG-based command of the movement of a robotic arm or wheelchair in HMI applications (Postelnicu et al. 2011; Ramli et al. 2015). We intend in the future to extend this work to the problem of feature selection for the peak detection algorithm, so as to optimize the selection of the most salient peak features, with an aim to improve further the performance of peak detection.
